# Long Noncoding RNA *LOC100129973* Suppresses Apoptosis by Targeting *miR-4707-5p* and *miR-4767* in Vascular Endothelial Cells

**DOI:** 10.1038/srep21620

**Published:** 2016-02-18

**Authors:** Wei Lu, Shu Ya Huang, Le Su, Bao Xiang Zhao, Jun Ying Miao

**Affiliations:** 1 Shandong Provincial Key Laboratory of Animal Cells and Developmental Biology, School of Life Science, Shandong University, Jinan 250100, China; 2 Institute of Organic Chemistry, School of Chemistry and Chemical Engineering, Shandong University, Jinan 250100, China; 3 The Key Laboratory of Cardiovascular Remodeling and Function Research, Chinese Ministry of Education and Chinese Ministry of Health, Shandong University Qilu Hospital, Jinan, 250012, China

## Abstract

Accumulating evidence has demonstrated that long non-coding RNAs (lncRNAs) are key regulators of multiple biological processes by altering gene expression at various levels. Apoptosis in vascular endothelial cells (VECs) is closely linked to numerous cardiovascular diseases, such as arteriosclerosis, thrombus formation and plaque erosion. However, studies on lncRNAs in the cardiovascular system are just beginning. And thus far, no anti-apoptosis lncRNAs have been identified in VECs. Here, we focused on the anti-apoptosis roles of lncRNAs in the serum and FGF-2 starvation-induced apoptosis of VECs. Using microarray analysis, we found a novel lncRNA *LOC100129973* which acted as an apoptosis inhibitor in VECs. Through sponging *miR-4707-5p* and *miR-4767*, lncRNA *LOC100129973* upregulated the expression of two apoptosis repressors gene, Apoptosis *Inhibitor 5* (*API5*) and *BCL2 like 12* (*BCL2L12*), and thus alleviated the serum and FGF-2 starvation-induced apoptosis in VECs. This evidence suggests that lncRNA *LOC100129973* is an attractive target to improve endothelial function and for therapy of apoptosis related cardiovascular diseases.

Long noncoding RNAs (lncRNAs) are defined as non-protein coding transcripts longer than 200 nucleotides without significant protein-coding potential. They constitute a large portion of mammalian transcriptome, since only ~2% of the mammalian genome is composed of genes that encode proteins^1^. LncRNAs could regulate the expression of genes at the epigenetic, transcriptional and post-transcriptional levels^2^,^3^,^4^. They play important roles in multiple physiological processes such as differentiation, proliferation, apoptosis, invasion and reprogramming of induced pluripotent stem cells^5^,^6^,^7^,^8^ by several regulatory mechanisms such as interacting with chromatin-modifying enzymes, RNA processing, structural scaffolds and so on^9^,^10^,^11^. In addition, the ability of lncRNAs to function as competing endogenous RNA (CeRNA) was first demonstrated in muscle differentiation^5^.

Vascular endothelial cells (VECs), which lie in the innermost of blood vessels, are vulnerable to stimulus. Apoptosis in VECs is closely linked to numerous cardiovascular diseases such as arteriosclerosis, thrombus formation and plaque erosion etc.^12^. Formerly, the investigation on the mechanisms of apoptosis mainly focused on the protein-coding genes. Recently, lncRNAs have attracted more and more interest^13^,^14^,^15^. Yet, there are no reports about apoptosis-related lncRNA in VECs.

Ischemia is a cardiovascular disease generally caused by atherosclerosis or thrombosis^16^,^17^, and is associated with apoptosis of VECs due to deficiency of survival growth factors^18^,^19^. In our previous work, human umbilical vein endothelial cells (HUVECs) were cultured under the serum and FGF-2-deprived condition to simulate the *in vivo* ischemic condition. We found that a small molecule, 6-amino-2,3-dihydro-3-hydroxymethyl-1,4-benzoxazine (ABO), elevated the viability of HUVECs in the absence of serum and FGF-2^20^. Moreover, it was demonstrated that ABO effectively inhibited oxLDL-induced apoptosis of VECs^21^ and atherosclerosis in ApoE^−/−^ mice^22^. These data suggest that ABO is an appropriate molecule for finding new factors that inhibit VEC apoptosis.

In this study, we aimed to find new factors which repress the serum and FGF-2 starvation-induced apoptosis of VECs by using ABO and microarray. Fortunately, we noticed that lncRNA *LOC100129973* was significantly increased by ABO treatment. Furthermore, we demonstrated that through sponging *miR-4707-5p* and *miR-4767*, lncRNA *LOC100129973* upregulated two apoptosis repressors, Apoptosis Inhibitor 5 (API5) and BCL2 like 2 (BCL2L12), and thus suppressed the serum and FGF-2 starvation-induced apoptosis in HUVECs.

## Results

### Long noncoding RNA *LOC100129973* was upregulated by ABO treatment in HUVECs

Our previous data suggested that ABO is an appropriate molecule for finding new factors which could inhibit VEC apoptosis^20^,^21^,^22^,^23^,^24^. By morphological observation, AO staining and TUNEL assay, we confirmed that ABO efficiently inhibited the serum and FGF-2 starvation-induced apoptosis in HUVECs (Supplemental Fig. S1). To gain insights into the possible anti-apoptosis factors in the serum and FGF-2 starvation-induced apoptosis of VECs, we detected the changed transcripts by using ABO and microarray. The microarray assay revealed 22 genes with modified expression, including 6 upregulated genes and 16 downregulated genes in response to 50 μM ABO (Supplementary Table S1). The most significantly upregulated transcript was *LOC100129973* (Gene ID: 100129973).

**Figure 1:**
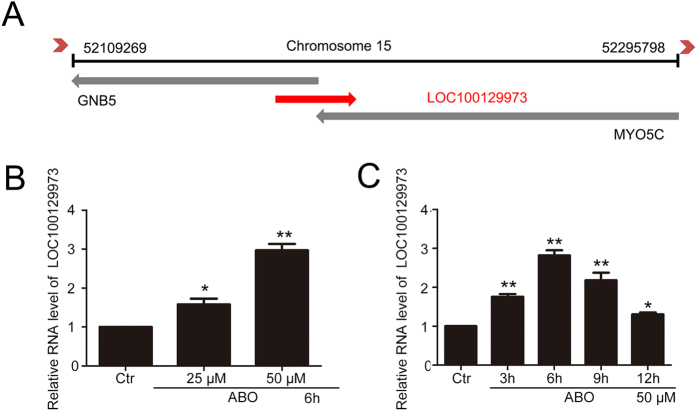
LncRNA *LOC100129973* was upregulated by ABO. (**A**) The basic information of lncRNA *LOC100129973* in human genome. (**B**) Quantified real-time PCR analysis of lncRNA *LOC100129973* expression treated with different concentrations of ABO treatment for 6 h in HUVECs deprived of serum and FGF-2. (**C**) Quantified real-time PCR analysis of lncRNA *LOC100129973* expression treated with 50 μM ABO for various times in HUVECs deprived of serum and FGF-2. Data are mean ± SEM. of three independent experiments. *P < 0.05, **p < 0.01 vs. control (Ctr). n ≥ 3.

*LOC100129973* is a validated long noncoding RNA (lncRNA), and the length of it is 1520 bp. This lncRNA is located in chromosome 15 (21.1) and antisense to *guanine nucleotide binding protein, beta 5* (*GNB5*) and *myosin VC* (*MYO5C*) (Fig. 1A). We next examined the expression pattern of lncRNA *LOC100129973* over a diverse panel of human cell types including HUVECs, hESCs, L-02 and human tumor cells such as A549, HeLa and PC3. Its expression was detected in all these human cells, while its expression is relative high in HUVECs (Supplemental Fig. S2). According to NCBI database, lncRNA *LOC100129973* was only found in Homo sapiens, Rhinopithecus roxellana and Macaca nemestrina. Hence, HUVECs is the ideal model for studying the role of lncRNA *LOC100129973*. Then, we validated the up-regulation of lncRNA *LOC100129973* using quantified real time RT-PCR (Fig. 1B,C). These results showed that in HUVECs, lncRNA *LOC100129973* was upregulated by ABO treatment in a dose- and time -dependent manner.

### LncRNA *LOC100129973* acted as an apoptosis repressor in HUVECs deprived of serum and FGF-2

**Figure 2:**
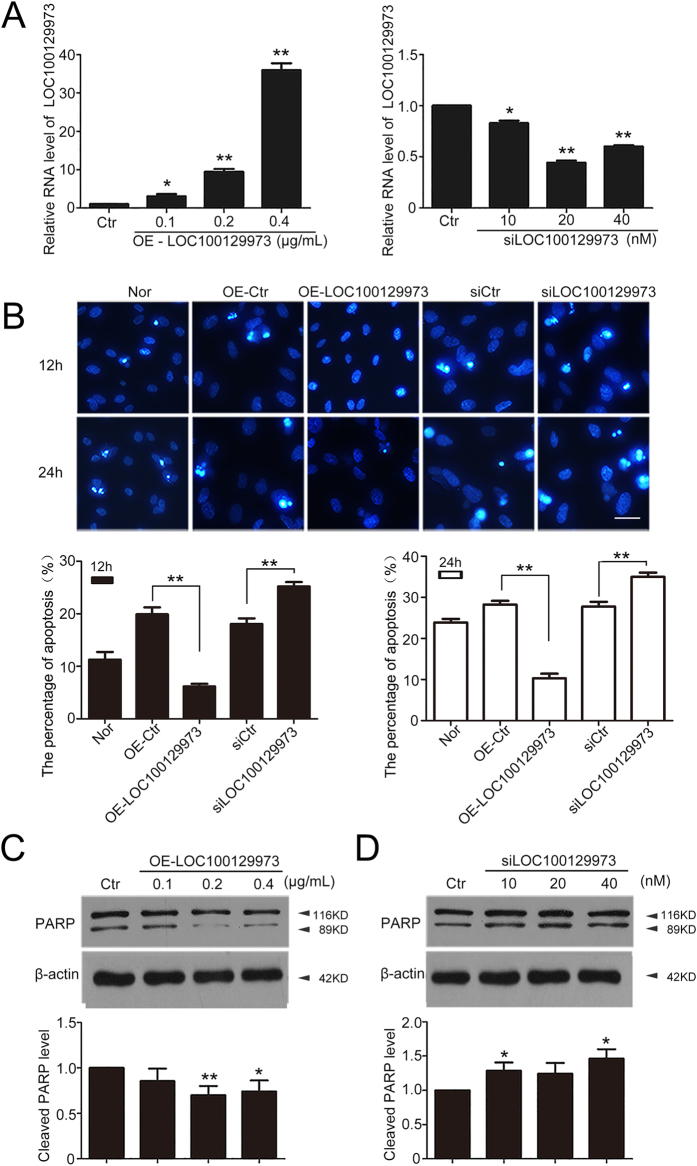
LncRNA *LOC100129973* suppressed the serum and FGF-2 starvation-induced apoptosis in HUVECs. (**A**) Quantified real-time PCR analysis of lncRNA *LOC100129973* overexpression and knock down efficiency. HUVECs were transfected with pcDNA3.1- *LOC100129973* at 0.1, 0.2, 0.4 μg/mL or si*LOC100129973* at 10, 20, 40 nM for 24 h and then starvation for another 24 h. (**B**) Hoechst 33258 staining of apoptotic HUVECs. Nor: M199 medium (with FGF-2 and serum); OE-Ctr: transfected with pcDNA3.1-empty vector; OE-*LOC100129973*: transfected with pcDNA3.1-*LOC100129973* at 0.2 μg/mL; siCtr: transfected with scramble RNA for negative control; si*LOC100129973*: transfected with si*LOC100129973* at 40 nM. After being transfected for 24 h, all the cells described above were deprived of serum and FGF-2 for another 12 or 24 h. Scale bar: 10 μm. Images are representative of at least 3 independent experiments. The percent of apoptosis was measured (%). (**C**,**D**) Western blot analysis of cleaved PARP protein level. HUVECs were transfected with pcDNA3.1-*LOC100129973* at 0.1, 0.2, 0.4 μg/mL or si*LOC100129973* at 10, 20, 40 nM for 24 h and starvation for another 24 h. The level of cleaved PARP was relative to that of β-actin. (Cropped, full-length blots are in Supplementary Fig. S4) Data are mean ± SEM. of three independent experiments. *P < 0.05, **p < 0.01 vs. control (Ctr). n ≥ 3.

To better understand the function of lncRNA *LOC100129973* in VECs, the full-length lncRNA *LOC100129973* was cloned into the pcDNA3.1 expression vector (pcDNA3.1- *LOC100129973*), and specific siRNAs against lncRNA *LOC100129973* (si*LOC100129973*) were designed and synthesized. HUVECs were transfected with pcDNA3.1- *LOC100129973* at 0.1, 0.2, 0.4 μg/mL or si*LOC100129973* at 10, 20, 40 nM. The efficiency of overexpression or knockdown was detected by quantified real time RT-PCR (Fig. 2A).

To clarify the roles of lncRNA *LOC100129973* in the serum and FGF-2 starvation-induced apoptosis of HUVECs, we examined cell viability, nuclear DNA condensation and cleaved PARP by SRB assay, Hoechst 33258 staining and western blot. Cell viability assay showed that enhanced lncRNA *LOC100129973* substantially increased cell viability, while the inhibition of lncRNA *LOC100129973* decreased it (Supplemental Fig. S3). Furthermore, overexpression of lncRNA *LOC100129973* inhibited the serum and FGF-2 starvation-induced apoptosis, whereas knockdown aggravated it (Fig. 2B). Moreover, lncRNA *LOC100129973* overexpression efficiently decreased cleaved PARP in HUVECs (Fig. 2C). And for knockdown of lncRNA *LOC100129973*, the cleaved PARP was evidently promoted (Fig. 2D). Collectively, our data showed that lncRNA *LOC100129973* acted as a repressor during the serum and FGF-2 starvation-induced apoptosis in HUVECs.

### LncRNA *LOC100129973* functioned as an endogenous sponge of *miR-4707-5p* and *miR-4767*

**Figure 3:**
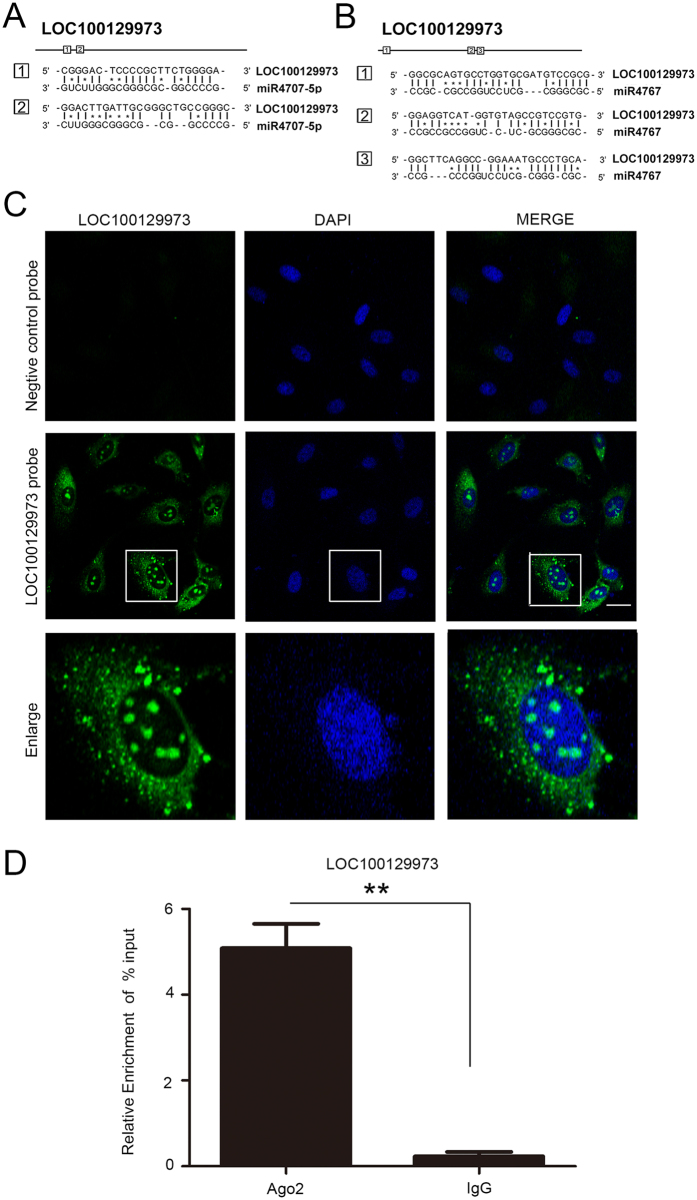
LncRNA *LOC100129973* might function as a miRNA sponge. (**A,B**) Potential sites targeted by *miR-4707-5p* and *miR-4767* in lncRNA *LOC100129973*. (**C**) RNA Fluorescent *in situ* hybridization of the LncRNA *LOC100129973* in HUVECs. The antisense probe was used as a negative control. Scale bar: 10 μm. (**D**) RNA-binding protein immunoprecipitation experiments were performed using Ago2 antibody in HUVECs. IgG was used as a negative control. Quantitative reverse transcription–polymerase chain reaction (qRT-PCR) was performed to detect pulled-down lncRNA *LOC100129973*. The lncRNA *LOC100129973* RNA level of input was set as 100%.

Recent studies have suggested that lncRNAs may act as endogenous sponge RNA to interact with miRNAs and influence the expression of these miRNAs^5^,^25^,^26^,^27^. To investigate the underlying mechanism of lncRNA *LOC100129973* action, we analyzed whether lncRNA *LOC100129973* could compete to bind with miRNAs as a miRNA-sponge by Micro Inspector (http://bioinfo.uni-plovdiv.bg/microinspector/). According to the analysis, there are two binding sites for *miR-4707-5p* and three for *miR-4767* (Fig. 3A,B). Meanwhile, the binding free energy of *miR-4707-5p* is the lowest and *miR-4767* is also very low: *miR-4707-5p* (−35.5) and *miR-4767* (−31.4). It is known that miRNAs are present in the cytoplasm in the form of miRNA ribonucleoprotein complexes (miRNPs) that also contain Ago2, the core component of the RNA-induced silencing complex (RISC)^28^,^29^. By RNA-fluorescence *in situ* hybridization (RNA-FISH), lncRNA *LOC100129973* was found to be distributed in both nucleus and cytoplasm of HUVECs (Fig. 3C). Moreover, lncRNA *LOC100129973* was preferentially enriched in Ago2-containing miRNPs relative to control immunoglobulin G (IgG) immunoprecipitates (Fig. 3D). Therefore, we speculated that lncRNA *LOC100129973* competed to bind with *miR-4707-5p* and *miR-4767* as a miRNA-sponge, and then regulated the expression of miRNA targets in HUVECs.

**Figure 4:**
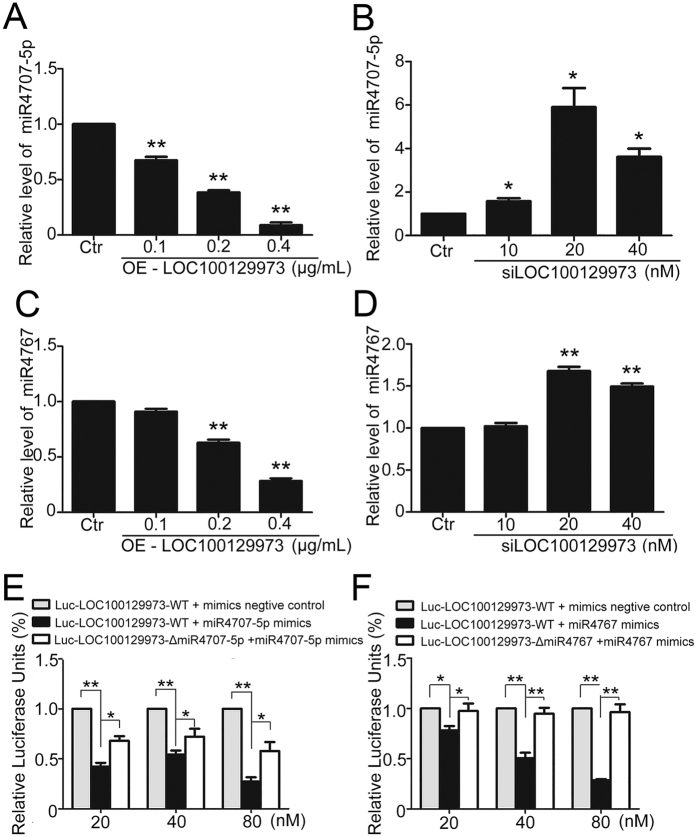
LncRNA *LOC100129973* regulated the expression of *miR-4707-5p* and *miR-4767* by directly binding with them. (**A**,**B**) miRNA quantified real-time PCR analysis of *miR-4707-5p* expression after lncRNA *LOC100129973* overexpression or knock down for 24 h and starvation for another 24 h in HUVECs. (**C**,**D**) miRNA quantified real-time PCR analysis of *miR-4767* expression after lncRNA *LOC100129973* overexpression or knock down for 24 h and starvation for another 24 h in HUVECs. (**E**) Luc-*LOC100129973*-WT or Luc-*LOC100129973*-Δ*miR-4707-5p* plasmids were co-transfected into HEK293T cells with 20, 40, 80 nM *miR-4707-5p* mimics or scrambled miRNA for 24 h and luciferase activity was measured. (**F**) Luc-*LOC100129973*-WT or Luc-*LOC100129973*-Δ*miR-4767* plasmids were co-transfected into HEK293T cells with 20, 40, 80 nM *miR-4767* mimics or scrambled miRNA for 24 h and luciferase activity was measured. Data are mean ± SEM. of three independent experiments. *P < 0.05, **p < 0.01 vs. control (Ctr). n ≥ 3.

After overexpression or knockdown of lncRNA *LOC100129973* for 24 h and starvation for another 24 h, we evaluated the RNA levels of *miR-4707-5p* and *miR-4767* in HUVECs. When lncRNA *LOC100129973* expression was enhanced, the RNA levels of *miR-4707-5p* and *miR-4767* were decreased in a dose-dependent manner (Fig. 4A,C); whereas, they were increased in a dose-dependent manner after lncRNA *LOC100129973* RNA level was declined (Fig. 4B,D). So lncRNA *LOC100129973* negatively regulated the RNA levels of *miR-4707-5p* and *miR-4767* in HUVECs.

To verify whether lncRNA *LOC100129973* act as a miRNA-sponge to direct-negatively regulate *miR-4707-5p* and *miR-4767* RNA levels, we constructed three dual-luciferase miRNA target expression constructs: (1) Luc-*LOC100129973*-WT; (2) Luc-*LOC100129973*-Δ*miR-4707-5p* (mutated on the putative *miR-4707-5p* sites); (3) Luc-*LOC100129973*-Δ*miR-4767* (mutated on the putative *miR-4767* sites). Luciferase assays revealed that the mimics of *miR-4707-5p* and *miR-4767* suppressed the luciferase activity of Luc-*LOC100129973*-WT in a dose-dependent manner, respectively (Fig. 4E,F). However, compared to Luc-*LOC100129973*-WT, *miR-4707-5p* mimics had less effect on Luc-*LOC100129973*-*ΔmiR-4707-5p* (Fig. 4E). Likewise, *miR-4767* mimics had less effect on Luc-*LOC100129973*-Δ*miR-4767* (Fig. 4F). Taken together, it suggested that lncRNA *LOC100129973* downregulated the RNA levels of *miR-4707-5p* and *miR-4767* through directly binding to them.

### *miR-4707-5p* and *miR-4767* promoted apoptosis by targeting and downregulating two apoptosis inhibitors, API5 and BCL2L12, respectively

**Figure 5:**
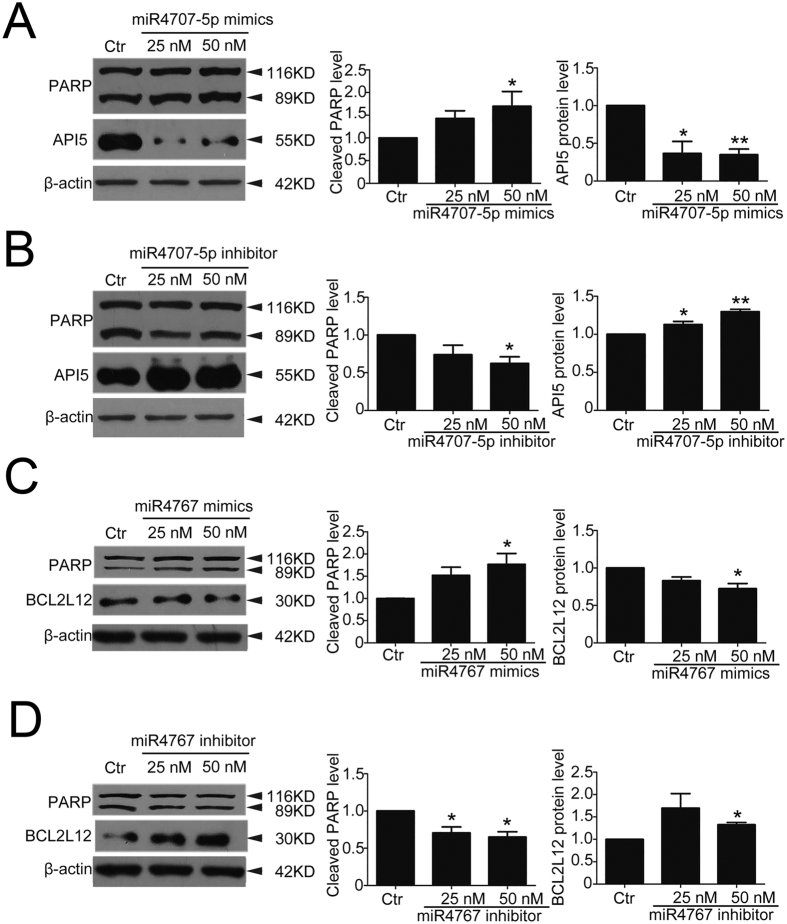
m*iR-4707-5p* and *miR-4767* promoted the serum and FGF-2 starvation-induced apoptosis and downregulated the protein of API5 and BCL2L12 in HUVECs. (**A**,**B**) Western blot analysis of cleaved PARP and API5 protein levels after transfection with *miR-4707-5p* mimics (**A**) or inhibitor (**B**) for 24 h and serum starvation for another 24 h in HUVECs. (Cropped, full-length blots are in Supplementary Fig. S7) (**C**,**D**) Western blot analysis of cleaved PARP and BCL2L12 protein levels after transfection with *miR-4767* mimics (**C**) or inhibitor (**D**) for 24 h and serum starvation for another 24 h in HUVECs. (Cropped, full-length blots are in Supplementary Fig. S8) Data are mean ± SEM. of three independent experiments. *P < 0.05, **p < 0.01 vs. control (Ctr). n ≥ 3.

To address whether lncRNA *LOC100129973* represses apoptosis though *miR-4707-5p* and *miR-4767*, we investigated the roles of *miR-4707-5p* and *miR-4767* in apoptosis of HUVECs. HUVECs were transfected with the mimics or inhibitors of *miR-4707-5p* and *miR-4767* at 25 or 50 nM for 24 h and starvation for another 24 h, the efficiency of overexpression or knockdown was detected (Supplemental Fig. S5). The western blot results showed that the mimics of *miR-4707-5p* or *miR-4767* enhanced the cleaved PARP (Fig. 5A,C), while the inhibitors reduced the cleaved PARP (Fig. 5B,D). It indicated that *miR-4707-5p* and *miR-4767* promoted the serum and FGF-2 starvation-induced apoptosis in HUVECs.

**Figure 6:**
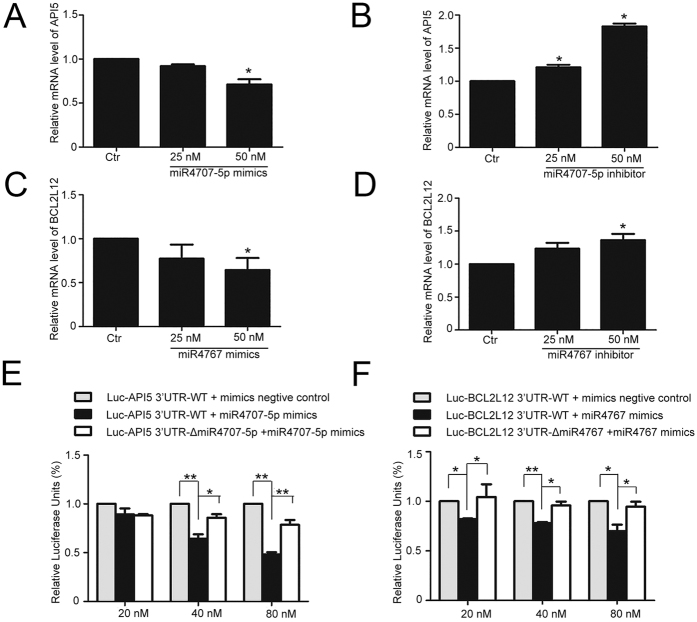
m*iR-4707-5p* and *miR-4767* directly - targeted to *API5* and *BCL2L12* and regulated the expression of them in HUVECs, respectively. (**A–D**) Quantified real-time PCR analysis of *API5*/*BCL2L12* mRNA levels after transfection with *miR-4707-5p*/*miR-4767* mimics (**A,C**) or inhibitor (**B,D**) for 24 h and starvation for another 24 h in HUVECs. (**E**) Luc-*API5* 3′UTR/Luc-*API5* 3′UTR -Δ*miR-4707-5p* plasmid was co-transfected into HEK293T cells with 20, 40, 80 nM *miR-4707-5p* mimics or scrambled miRNA for 24 h and luciferase activity was measured. (**F**) Luc-*BCL2L12* 3′UTR/Luc-*BCL2L12* 3′UTR-Δ*miR-4767* plasmid was co-transfected into HEK293T cells with 20, 40, 80 nM *miR-4767* mimics or scrambled miRNA for 24 h and luciferase activity was measured. Data are mean ± SEM. of three independent experiments. *P < 0.05, **p < 0.01 vs. control (Ctr). n ≥ 3.

For further exploring the possible molecular mechanism of *miR-4707-5p* and *miR-4767* in promoting apoptosis, we predicated the target genes of *miR-4707-5p* and *miR-4767* by miRDB, Targetscan and DIANA LAB. Among these potential targets, we concentrated on *Apoptosis Inhibitor 5 (API5)* and *BCL2 like 2 (BCL2L12)*, both of which inhibit cell apoptosis^30^,^31^,^32^,^33^. By using RNAhybrid software, we found that 3′UTR of *API5* contained the *miR-4707-5p*-prediction binding sites with the highest score. Likewise, 3′UTR of *BCL2L12* contains two *miR-4767*-prediction binding sites (Supplemental Fig. S6). Furthermore, we tested whether *miR-4707-5p* and *miR-4767* could regulate the expression levels of *API5* and *BCL2L12.* Enhanced RNA level of *miR-4707-5p* or *miR-4767* led to a reduction on the RNA level (Fig. 6A,C) and protein level (Fig. 5A,C) of *API5* or *BCL2L12* in HUVECs. In contrast, knockdown of endogenous *miR-4707-5p* or *miR-4767* induced an increase on the RNA level (Fig. 6B,D) and protein level (Fig. 5B,D) of *API5* or *BCL2L12* in HUVECs. These results showed that *miR-4707-5p* and *miR-4767* downregulated the expression of two apoptosis inhibitors, API5 and BCL2L12, respectively.

To verify whether *miR-4707-5p* and *miR-4767* directly target to *API5* and *BCL2L12* respectively, we constructed four dual-luciferase miRNA target expression constructs: (1) Luc-*API5* 3′UTR; (2) Luc-*API5* 3′UTRmut (mutated on the putative miR-*4707-5p* sites); (3) Luc-*BCL2L12* 3′UTR; (4) Luc-*BCL2L12* 3′UTRmut (mutated on the putative miR-4767 sites). Luciferase assays showed that *miR-4707-5p* mimics suppressed luciferase activity of Luc-*API5* 3′UTR in a dose-dependent manner, but not Luc-*API5* 3′UTRmut (Fig. 6E); meanwhile, the mimics of *miR-4767* also reduced luciferase activity of Luc-*BCL2L12* 3′UTR in a dose-dependent manner, but not Luc-*BCL2L12* 3′UTRmut (Fig. 6F). Collectively, these results suggested that *miR-4707-5p* and *miR-4767* induced apoptosis in HUVECs by directly targeting and decreasing the expression of two apoptosis inhibitors, API5 and BCL2L12, respectively.

### LncRNA *LOC100129973* promoted the expression of two apoptosis inhibitors, *API5* and *BCL2L12*, by sponging *miR-4707-5p* and *miR-4767*

**Figure 7:**
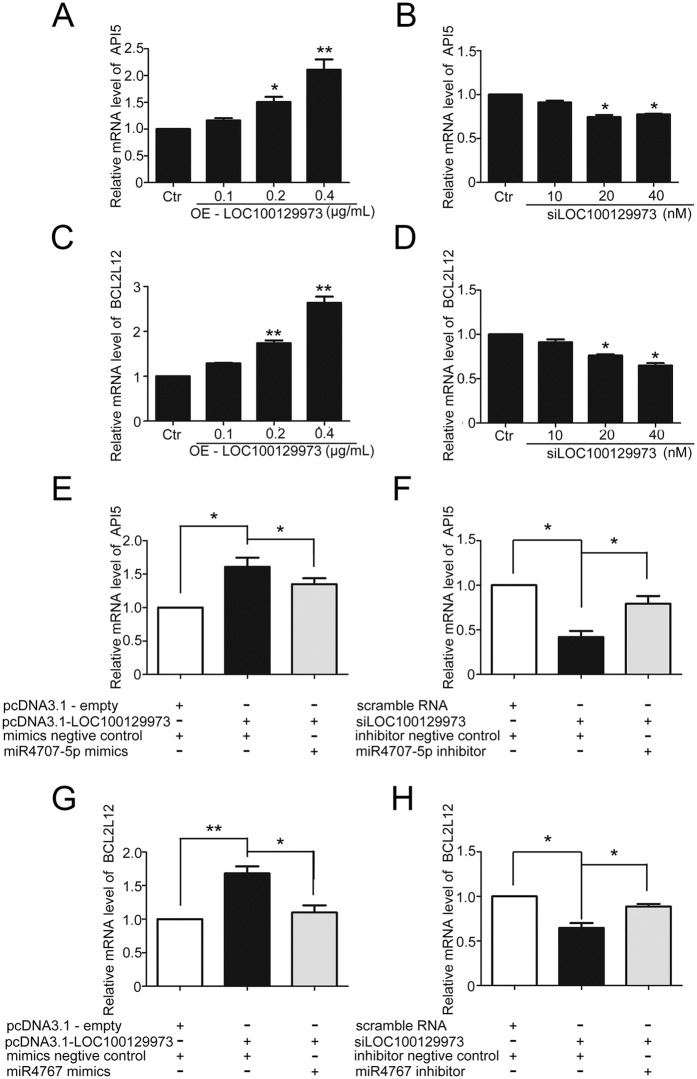
LncRNA *LOC100129973* positively regulated *API5* and *BCL2L12* expression mediated by *miR-4707-5p* and *miR-4767*. **(A–D**) After transfection with pcDNA3.1-*LOC100129973* plasmid or si*LOC100129973* for 24 h and serum starvation for another 24 h, quantified real-time PCR analysis of *API5* (**A,B**)/*BCL2L12* (**C,D**) expression in HUVECs were performed. (**E–H**) Quantified real-time PCR analysis of *API5*/*BCL2L12* expression after being cotransfected with pcDNA3.1-*LOC100129973* at 0.2 μg/mL and *miR-4707-5p*/*miR-4767* mimics or mimics negative control at 50 nM (**E,G**); Being cotransfected with si*LOC100129973* at 40 nM and *miR-4707-5p*/*miR-4767* inhibitor or inhibitor negative control at 50 nM (F and H) for 24 h, and followed with serum starvation for another 24 h. Data are mean ± SEM. of three independent experiments. *P < 0.05, **p < 0.01 vs. control (Ctr).

Given that lncRNA *LOC100129973* could function as an endogenous sponge of *miR-4707-5p* and *miR-4767*, which could reduce the expression of *API5* and *BCL2L12*, we supposed that lncRNA *LOC100129973* might regulate the expression of *API5* and *BCL2L12* through *miR-4707-5p* and *miR-4767*. We observed that the mRNA levels of two apoptosis inhibitors, *API5* and *BCL2L12*, were increased after enforced lncRNA *LOC100129973* expression in HUVECs (Fig. 7A,C); conversely, they were decreased by transfection with si*LOC100129973* (Fig. 7B,D).

Furthermore, we investigated the roles of *miR-4707-5p* and *miR-4767* in the lncRNA *LOC100129973*-induced upregulation of *API5* and *BCL2L12*. Enhanced RNA levels of *miR-4707-5p* or *miR-4767* in HUVECs with lncRNA *LOC100129973* overexpression, led to decrease the expression of *API5* or *BCL2L12* (Fig. 7E,G). Conversely, by reducing RNA levels of *miR-4707-5p* or *miR-4767* in HUVECs with lncRNA *LOC100129973* knockdown, the expression level of *API5* or *BCL2L12* was increased (Fig. 7F,H). Taken together, our data demonstrated that, through sponging *miR-4707-5p* and *miR-4767*, lncRNA *LOC100129973* was able to positively regulate the expression of *API5* and *BCL2L12*, and thus alleviate the serum and FGF-2 starvation-induced apoptosis in HUVECs.

## Discussion

**Figure 8:**
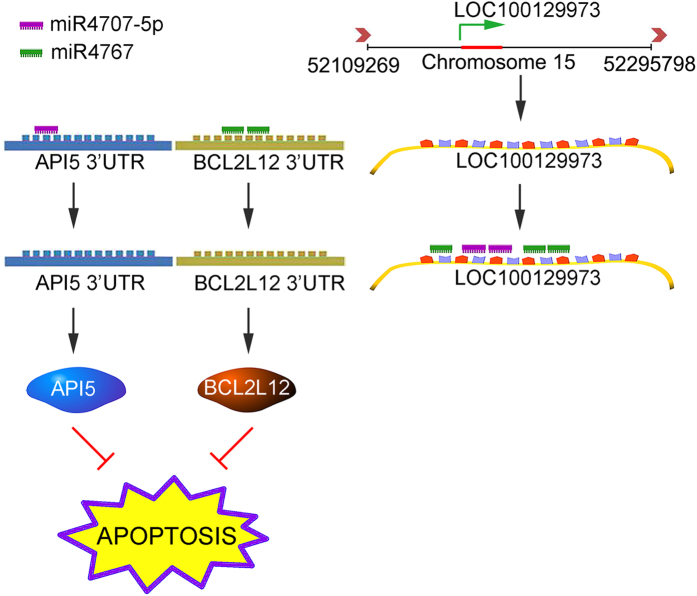
Schematic diagram of lncRNA *LOC100129973*-related regulatory mechanism in endothelial cell apoptotic signaling. LncRNA *LOC100129973*, which functions as a ceRNA to bind with *miR-4707-5p* and *miR-4767*, regulates the expression of their targets (*API5* and *BCL2L12*), and further controls apoptosis in HUVECs.

In this study, we first found that lncRNA *LOC100129973* was significantly increased by ABO treatment and demonstrated that lncRNA *LOC100129973* was an apoptosis inhibitor. Furthermore, lncRNA *LOC100129973* negatively regulated *miR-4707-5p* and *miR-4767* by directly binding with them. The *miR-4707-5p* and *miR-4767* aggravated apoptosis by targeting the 3′UTR of two apoptosis inhibitors, *API5* and *BCL2L12*, respectively. Hence, lncRNA *LOC100129973* increased the expression of *API5* and *BCL2L12* by sponging *miR-4707-5p* and *miR-4767*, and thus inhibited the serum and FGF-2 starvation-induced apoptosis in HUVECs (Fig. 8).

It has been well demonstrated that lncRNAs take part in multiple human diseases through altering gene expression at various levels. Currently uncovered functions of lncRNAs are classified as the followings: (1) Imprinting; (2) Scaffold/Guide for Epigenetic and Transcription Factors; (3) Enhancer Activation; (4) Molecular Sponges^34^. Obviously, lncRNA *LOC100129973* is a distinct molecular sponge, which could sequester *miR-4707-5p* and *miR-4767* and regulate the expression of their targets.

Cardiovascular diseases are one of the major causes of death worldwide and the morbidity is increasing year by year^35^. Although studies on lncRNAs in the cardiovascular system are just beginning, more and more reports have shown that lncRNAs are involved in multiple cardiovascular diseases^34^. For atherosclerosis, lncRNA *H19* and *ANRIL* play roles in the pathologic mechanism of atherosclerosis^36^,^37^. LncRNA *Kcnq1ot1* regulated *Kcnq1* levels in the embryonic heart by altering chromatin flexibility and access to enhancers. However, among these researches, studies of lncRNAs in the VECs are bare and in infancy^34^. As yet, there are no reports about apoptosis-related lncRNA which could function as a molecular sponge in VECs. Therefore, lncRNA *LOC100129973*, acting as a competing endogenous RNA, is a novel long noncoding RNA which suppresses apoptosis in VECs by upregulating the expression of *API5* and *BCL2L12*.

So far, there are no studies on *miR-4707-5p* and *miR-4767* functions. By using bioinformatics prediction analysis and luciferase activity assay, we indicated that *API5* and *BCL2L12* were targets of *miR-4707-5p* and *miR-4767*, respectively.

*Apoptosis Inhibitor 5* (*API5*), also known as *ACC-11* or *EIF*, was initially identified as a nuclear protein for its anti-apoptotic function. Overexpression of *API5* prevents apoptosis after serum and FGF-2 starvation^38^. API5 is able to bind with FGF-2, further to form FGF-2/FIF complex *in vivo*^39^. As a CeRNA, lncRNA *LOC100129973* could compete with *miR-4707-5p* to control *API5* expression (Fig. 8). Hence, subdued VEC apoptosis mediated by *LOC100129973* may be partially resulted from the enhanced FGF-2 signaling.

*BCL2 like-12* (*BCL2L12*), encoding a BCL2-like proline-rich protein, is a member of the Bcl-2 family of apoptosis-regulating proteins^40^. BCL2L12 locates in both cytoplasm and nucleus^41^. Nuclear BCL2L12 physically interacts with p53 to form a complex, and thus eliminates the tumor suppression of p53^41^,^42^. BCL2L12 inhibits post-mitochondrial apoptosis signaling - caspase-3/7 via distinct mechanisms^43^. For caspase 7, BCL2L12 directly interacts with it and then neutralizes its function^44^. For caspase 3, BCL2L12-induced transcriptional upregulation of the small heat shock protein αB-crystallin is instrumental to neutralization of caspase 3 activation^43^,^45^. As a CeRNA, lncRNA *LOC100129973* could compete with *miR-4767* to control *BCL2L12* expression (Fig. 8). Therefore, in HUVECs, decreased apoptosis mediated by lncRNA *LOC100129973* may be partially resulted from alleviating activation of caspase-3/7 and p53.

In summary, as shown in Fig. 8, with decoy activity of sequestering *miR-4707-5p and miR-4767*, lncRNA *LOC100129973* promotes the expression of the *API5* and *BCL2L12*, and thus suppresses apoptosis. Therefore, lncRNA *LOC100129973* is an attractive target to improve endothelial function and for therapy of apoptosis related cardiovascular diseases.

## Methods

### Ethics statement

In this study, all subjects filled out a questionnaire which included their informed consent. The human subjects and all experimental procedures were performed in accordance with the ARRIVE guidelines^46^ and approved by the ethics committee in Shandong University, China.

### Cell Culture

Human umbilical vein endothelial cells (HUVECs) were isolated from umbilical cords as described^47^, then cultured in M199 medium (Gibco, 31100-035) with 10% (v/v) fetal bovine serum (Hyclone, SV30087.02) and 10 IU/mL fibroblast growth factor 2 (FGF-2) in a humidified incubator at 37 °C with 5% CO_2_. Cells at not more than passage 10 were used for experiments. HEK293T cells were obtained from the Cell Bank of the Chinese Academy of Sciences (Shanghai, China) and were grown in DMEM (Gibco) with 8% fetal bovine serum (FBS; Gibco), penicillin (50 U/ml) and streptomycin (50 μg/ml) (Gibco, 10378-016).

### Cell apoptosis assay

The cells treated as mentioned ways for 12 or 24 h were stained with acridin orange (AO, Fluka) for 5 min or Hochest33258 (10 μg/mL) for 15 min 37 °C. Stained cells were washed twice with PBS and then observed under a laser scanning confocal microscope (Leica, DMIRE2, Wetzlar, Germany) or an Olympus (Japan) BH-2 fluorescence microscope. Cells were regarded as apoptotic if nuclei were much brighter or showed condensed chromatin and nuclear fragmentation.

Terminal deoxynucleotidyl transferase-mediated dUTP nick end labeling (TUNEL) was used to detect DNA fragmentation and to calculate apoptotic ratio. TUNEL was carried out following the manufacturer’s instructions (G3250 DeadEndTM Fluorometric TUNEL System, Promega, USA)^48^. For negative controls, TdT enzyme was not included in the incubation buffer. The apoptotic index was quantified by calculating the number of positive TUNEL cells from total 500 cells in five random microscopic fields.

### LncRNA microarray analysis

HUVECs were treated with 50 μM ABO or 0.05% dimethyl sulfoxide (DMSO) in basal M199 medium for 6 h. Then total RNA samples were extracted with Trizol reagent (Invitrogen, 15596018). Agilent human long noncoding RNA 4 × 180K microarray platform was used in this study and lncRNA microarray analysis was performed by CapitalBio Corporation.

### Quantitative real-time PCR

Total RNA was extracted from HUVECs by the Trizol reagent method (Invitrogen, Carlsbad, CA, USA) and underwent reverse transcription and quantitative RT-PCR (Roche, Light Cycler 2.0 system) with the primer pair sequences for genes (Supplementary Table S2). The reverse transcription step involved use of the PrimeScript RT reagent kit with gDNA Eraser (DRR047, TAKARA). For miRNAs, *miR-4707-5p* and *miR-4767* were converted into cDNA with the TaqMan MicroRNA Reverse Transcription Kit (Applied Biosystems, PN4366596). The expression of mature *miR-4707-5p* and *miR-4767* in HUVECs were quantified by the KAPA SYBR FAST qPCR Kit (Kapa Biosystems, KK4601). Small RNA *U6* was used as internal control for small RNAs. Relative gene expression of *miR-4707-5p* or *miR-4767* was normalized to *U6*. Quantitative RT-PCR reactions involved use of SYBR Premix Ex Taq (Tli RNaseH Plus) and were carried out in a 20 μl volume with 10 μl of 2X SYBR Green I, 0.4 μl sense primer, 0.4 μl antisense primer, 2 μl cDNA template and 7.2 μl distilled water. Relative gene expression was normalized to that of a housekeeping gene (β - actin). The levels of expressed genes were measured by the 2^−ΔΔCt^ method with MxPro 4.00 (Stratagene).

### Transient transfection with plasmid in HUVECs

pcDNA3.1-*LOC100129973* construct was generated by inserting the full length of lncRNA *LOC100129973* into the pcDNA3.1 vector. HUVECs were cultured to full confluent prior to transfection in basic M199 medium, and transient transfection was performed using Lipofectamine 2000 (Invitrogen, 11668-019) transfection reagent according to the manufacturer’s instructions. Cells were transfected with pcDNA3.1- empty as control.

### Transient transfection with siRNA or miRNA mimics/inhibitor in HUVECs

Specific siRNAs against lncRNA *LOC100129973* were synthesized by Invitrogen. Scramble siRNA was used as a control (Santa Cruz, sc-37007). RNA mimics and inhibitor for *miR-4707-5p/miR-4767* were designed and purchased from Invitrogen. Corresponding Negative Control (NC) was designed and purchased from Invitrogen. Cells at 70% confluence were transfected with siRNA or miRNA mimics/inhibitor by Lipofectamine 2000 (Invitrogen, 11668–019) transfection reagent according to the manufacturer’s instructions.

### Western blot analysis

Treated HUVECs were lysed in protein lysis buffer (Beyotime, P0013). Protein content was determined by use of the BCA Protein Assay Kit (Beyotime, P0011). Proteins were separated by 12% or 9% SDS-PAGE and transferred to PVDF membrane (Millipore, IPVH00010), which was incubated with primary antibodies for PARP (Cell Signaling, 9542L), Apoptosis Inhibitor 5 (API5; Abcam, ab65836), BCL2-like 12 (BCL2L12, Abcam, ab108346) and ACTB (Sigma, 122M4782) at 4 °C overnight and detected with corresponding horseradish peroxidase-conjugated secondary antibody (1:10000) at room temperature for 1 h. The membranes were incubated with Immobilon Western Chemiluminescent HRP Substrate (Millipore, WBKLS0500) for 5 min at room temperature and exposed to X-ray film (Kodak). The relative protein content was analyzed by ImageJ software and normalized to loading controls.

### Luciferase reporter transfection and dual luciferase activity assay

HEK293T cells were seeded into 96-well plates at 8000 cells per well for cultured overnight, then co-transfected with plasmids of dual-luciferase (containing firefly and Renilla luciferase) reporters and miRNA mimics or NC (final concentration, 20, 40 or 80 nM) with Lipofectamine 2000 (Invitrogen, 11668–019) for 24 h. Dual luciferase activity was measured by the Dual-Glo Luciferase Assay System (Promega, E2920). After reagent was added, firefly luciferase or Renilla luciferase activity was measured by VICTOR X2 Multilabel Plate Reader (PerkinElmer, USA). Firefly luciferase activity was normalized to that of Renilla.

### RNA fluorescent *in situ* hybridization (RNA-FISH)

LncRNA *LOC100129973* subcellular localization in HUVECs was detected by use of a FISH kit (Roche Applied Science, Germany). Briefly, HUVECs were fixed in 4% paraformaldehyde, then prehybridized with hybridization solution and incubated with a digoxigenin-labeled LncRNA *LOC100129973* probe. The antisense probe was used as a negative control. Cell nuclei were stained with 4′,6-diamidino-2-phenylindole (DAPI) for 5 min at room temperature. Fluorescence images were obtained by use of an LSM 700 Confocal Laser Scanning Microscope (Carl Zeiss).

### RNA-binding protein immunoprecipitation (RIP) assay

RNA immunoprecipitation (RIP) experiments were performed with the Magna RIP RNA-Binding Protein Immunoprecipitation Kit (Millipore, 17–701) following the manufacturer’s instructions. The RIPAb^+^ Ago2 antibody (Millipore, 03–110) was used in RIP and mouse IgG was used as a negative control. The PCR primers for lncRNA *LOC100129973* are listed in Supplementary Table S2.

### Statistical analysis

Data are presented as mean ± SEM and analysis involved use of GraphPad Prism 5. Images were processed by Adobe Photoshop CS5 (Adobe, San Jose, USA). P < 0.05 was considered statistically significant.

## Additional Information

**How to cite this article**: Lu, W. *et al.* Long Noncoding RNA *LOC100129973* Suppresses Apoptosis by Targeting *miR-4707-5p* and *miR-4767* in Vascular Endothelial Cells. *Sci. Rep.* **6**, 21620; doi: 10.1038/srep21620 (2016).

## Supplementary Material

Supplementary Information
