# Supplementary material for: Long Noncoding RNA LOC100129973 Suppresses Apoptosis by Targeting miR-4707-5p and miR-4767 in Vascular Endothelial Cells
Source: Sci Rep. 2016 Feb 18;6:21620. doi: 10.1038/srep21620 (PMC4757888; doi:10.1038/srep21620)
Supplement: Supplementary Information [file srep21620-s1.pdf]

Long Noncoding RNA *LOC100129973* Suppresses Apoptosis by Targeting *miR-4707-5p* and *miR-4767* in Vascular Endothelial Cells

Wei Lu[1], ShuYa Huang[1], Le Su[1], BaoXiang Zhao[2,*], JunYing Miao[1,3,*]

[1] Shandong Provincial Key Laboratory of Animal Cells and Developmental Biology, School of Life Science, Shandong University, Jinan 250100, China

[2] Institute of Organic Chemistry, School of Chemistry and Chemical Engineering, Shandong University, Jinan 250100, China

[3] The Key Laboratory of Cardiovascular Remodeling and Function Research, Chinese Ministry of Education and Chinese Ministry of Health, Shandong University Qilu Hospital, Jinan, 250012, China

*Correspondence to: Prof. JunYing Miao and Prof. BaoXiang Zhao, Institute of Developmental Biology, School of Life Science, Shandong University, Jinan 250100, China.

Fax: + 86 531 88565610; Tel.: + 86 531 88364929.

E-mail address: miaojy@sdu.edu.cn, bxzhao@sdu.edu.cn

Supplementary Information

Supplementary Figures

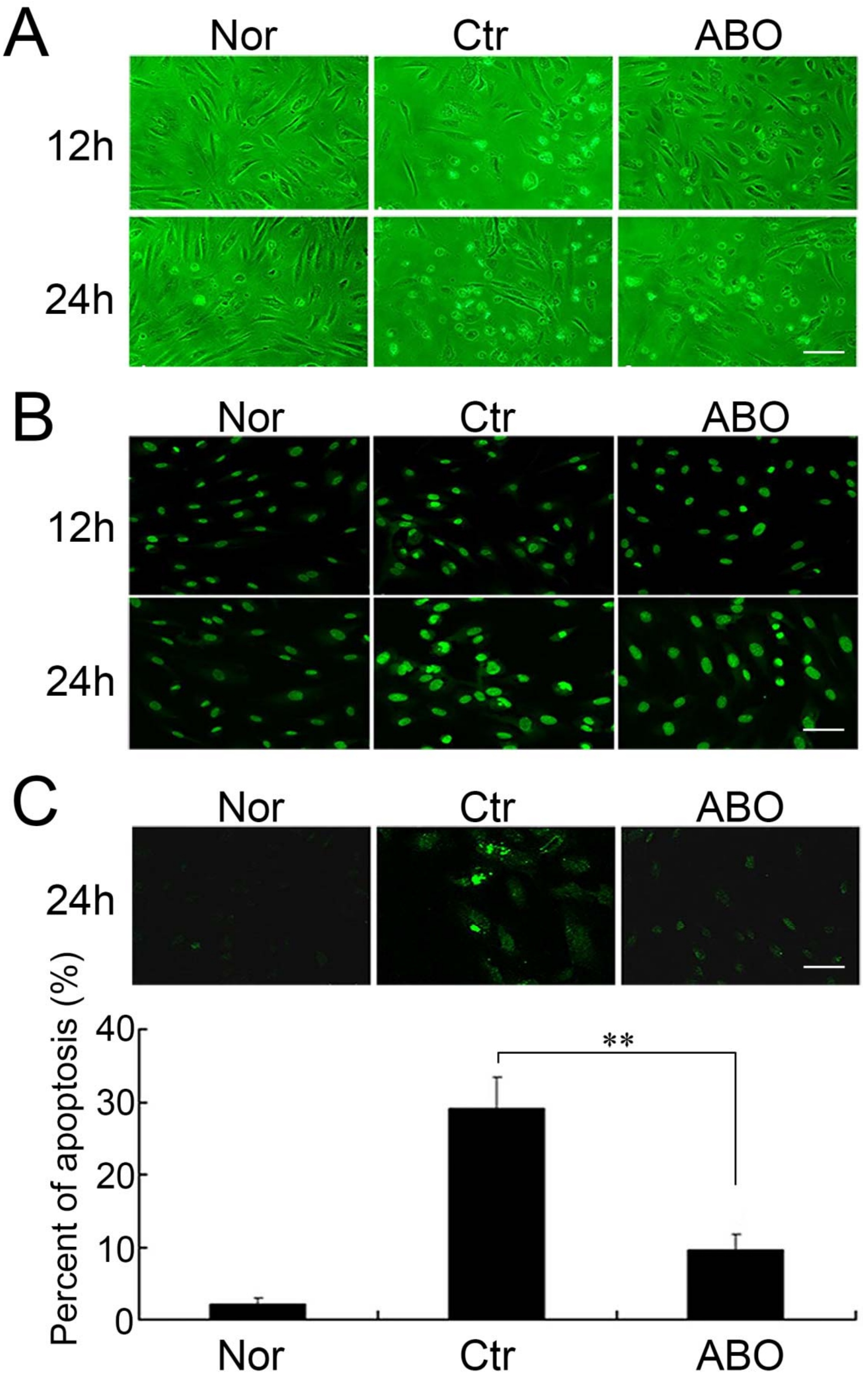

Figure S1 Effect of ABO on VEC apoptosis induced by deprivation of serum and FGF-2. When HUVECs reached to 80% confluent, treated in three ways: Nor: M199 medium (with FGF-2 and serum); Ctr: basal M199 medium with 0.05% dimethyl sulfoxide (DMSO); ABO: basal M199 medium with 50 μM ABO. (A) The morphological changes of the cells treated in the three ways as described above for 12 and 24h. Scale bar: 20 μm. Images are representative of at least 3 independent experiments. (B) The changes of nuclear fragmentation and chromatin condensation were observed by AO staining in the cells treated in the three ways as described above for 12 and 24h. Scale bar: 20 μm. Images are representative of at least 3 independent experiments. (C) DNA fragmentation detected by TUNEL assay in the cells treated in the three ways as described above for 24h. Scale bar: 20 μm. Images are representative of at least 3 independent experiments. The percent of apoptosis was measured (%). Data are mean ± SEM. of three independent experiments. *$P < 0.05$, ** $p < 0.01$ vs. control (Ctr). $n \geq 3$.

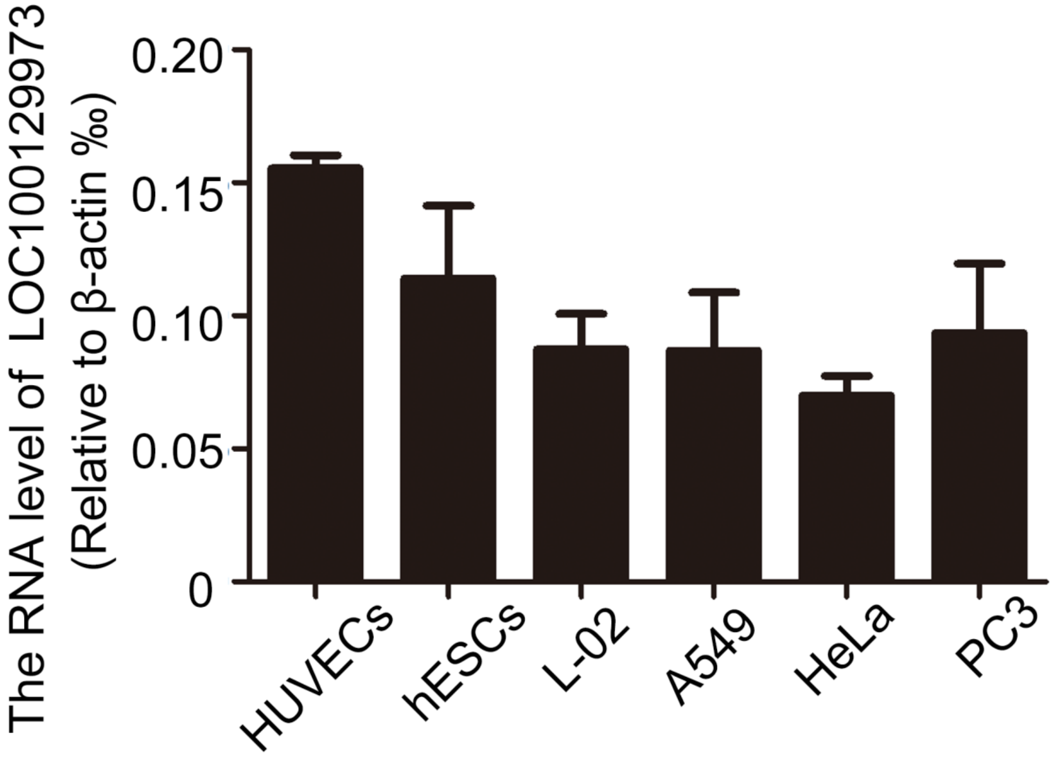

Figure S2 The cell-specific expression of lncRNA *LOC100129973*. Data are mean ± SEM. of three independent experiments.

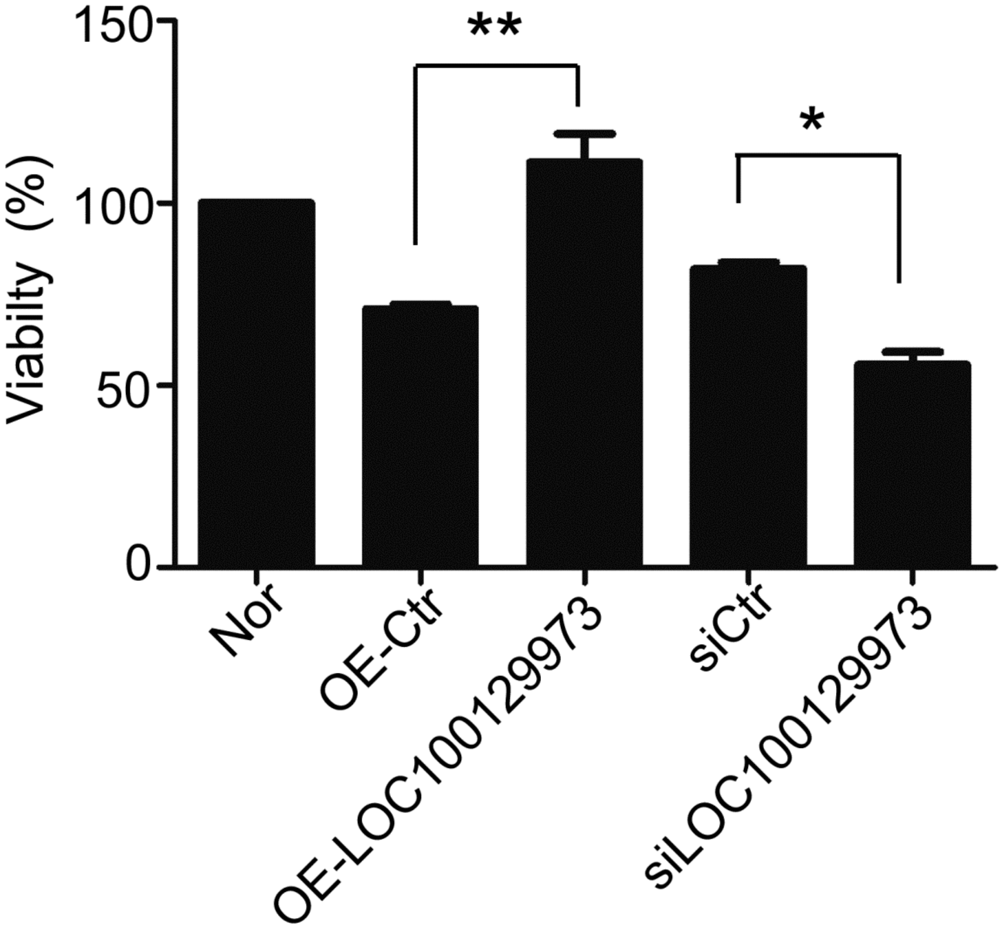

Figure S3 LncRNA *LOC100129973* positively regulated cell viability in HUVECs. Cells were seeded onto 96-well plates. Nor: M199 medium (with FGF-2 and serum); OE-Ctr: transfected with pcDNA3.1-empty vector; OE-*LOC100129973*: transfected with pcDNA3.1-*LOC100129973* at 0.2μg/mL; siCtr: transfected with scramble RNA for negative control; si*LOC100129973*: transfected with si*LOC100129973* at 40nM for 24h. After transfected for 24h, all the cells described above were deprived of serum and FGF-2 for another 24h. SRB assay was used to determine the cell viability.

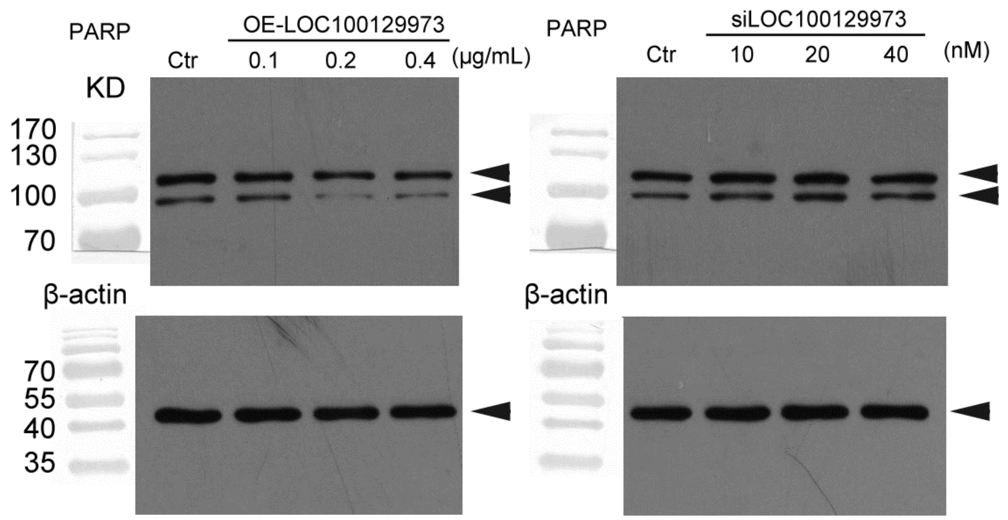

Figure S4 Uncropped blots probed with PARP and β-actin in Fiugre 2.

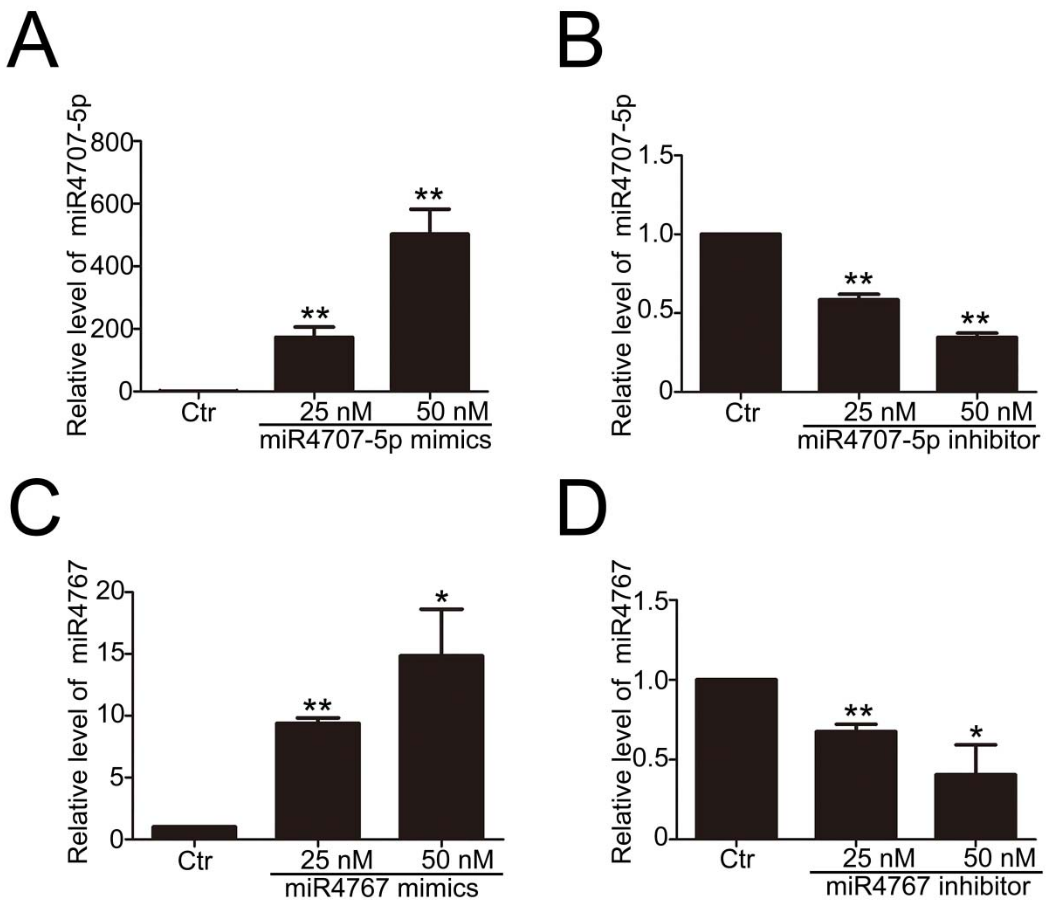

Figure S5 miRNA quantified real-time PCR analysis of *miR-4707-5p*/*miR-4767* after transfection with mimics and inhibitor at 25, 50nM for 24h, then serum starvation for another 24h in HUVECs. Data are mean ± SEM. of three independent experiments. $*P < 0.05$, $** p < 0.01$ vs. control (Ctr). $n \geq 3$.

A

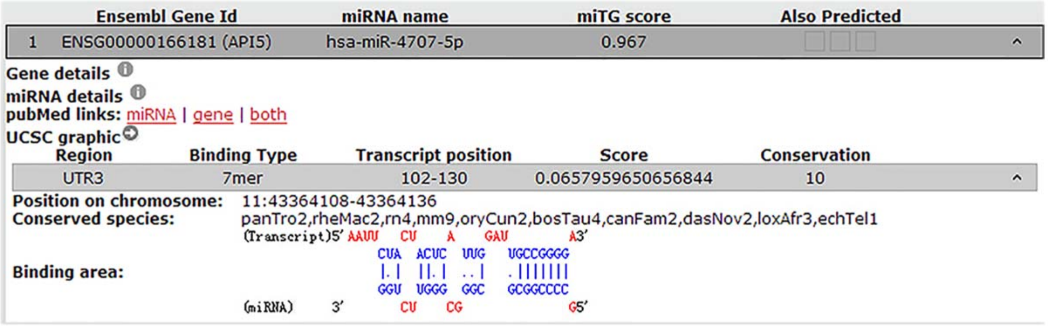

B

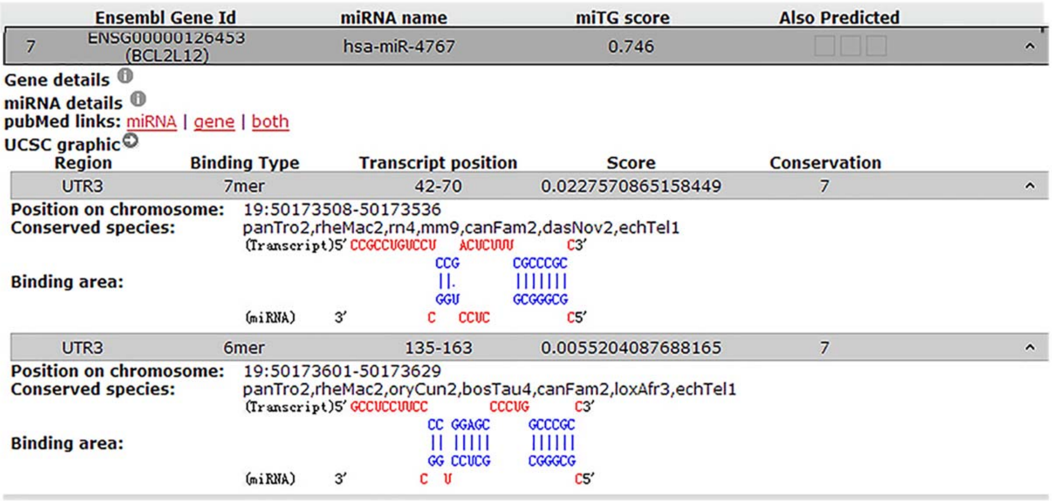

Figure S6 Predicted sites in 3'UTR of *API5* and *BCL2L12* targeted by *miR-4707-5p* and *miR-4767*. (RNAhybrid) (A) Potential sites in *API5* 3'UTR targeted by *miR-4707-5p*. (B) Potential sites in *BCL2L12* 3'UTR targeted by *miR-4767*.

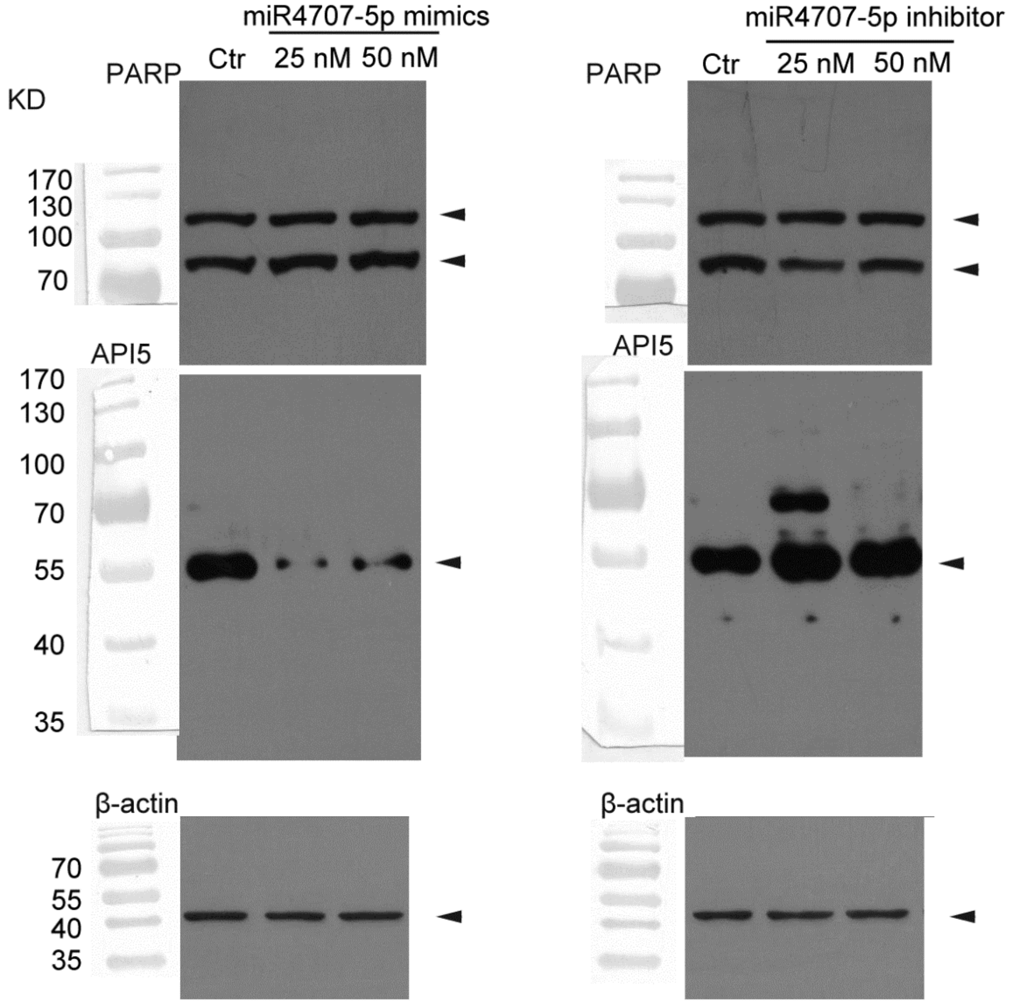

Figure S7 Uncropped blots probed with PARP, API5 and β-actin in Fiugre 5A and B.

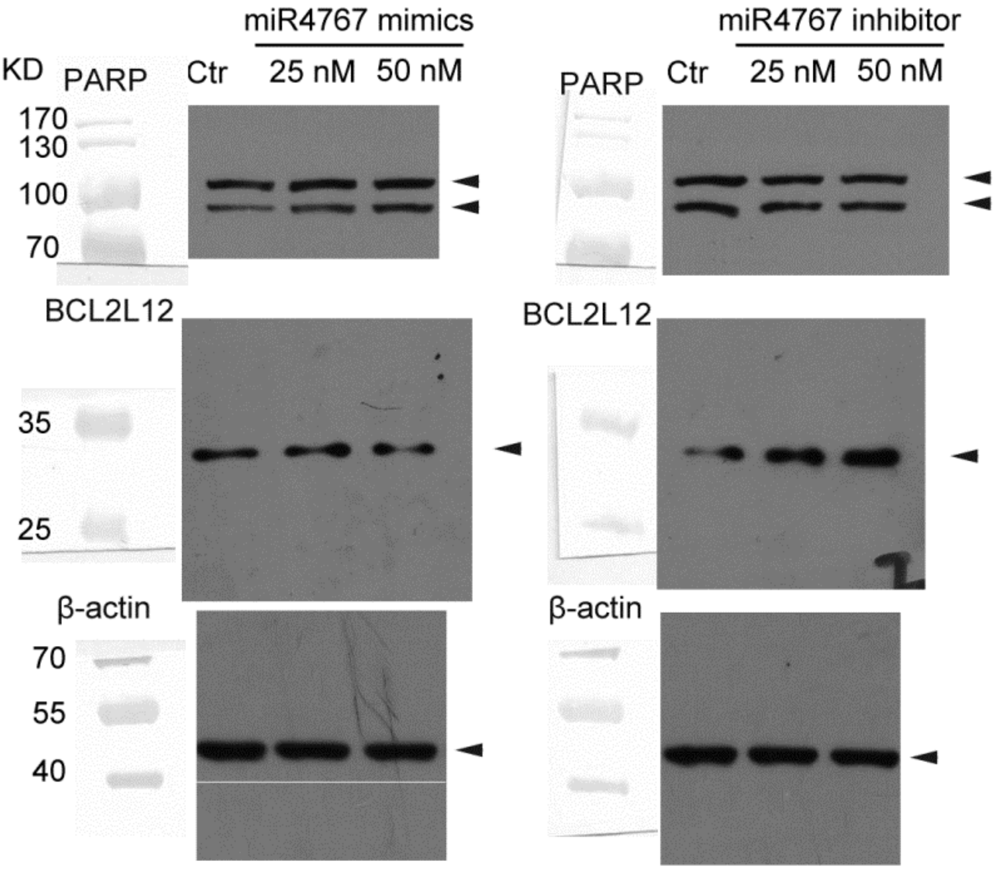

Figure S8 Uncropped blots probed with PARP, BCL2L12 and β-actin in Fiugre 5C and D.

Supplementary Tables

Table S1. Differentially expressed transcripts induced by 50 μM ABO for 6 h under serum and FGF-2 starvation.

Gene name	Cy5/(T) Intensity	Cy3/(C) Intensity	Ratio(T/C)
**Increased**			
LOC100129973	3778	1026	3.682
PDPK1	3403	1862	1.828
AK024714	1903	1047	1.818
ARMET	1947	1125	1.731
DKFZP566M1046	1774	1210	1.466
COQ4	1942	1334	1.456
**Decreased**			
AK023769	271	4088	0.066
ABCC3	302	4409	0.069
APEG1	139	1660	0.084
GIMAP5	527	3687	0.143
AK056982	592	2656	0.223
FGFBP1	382	1536	0.249
TEKT3	385	1536	0.251
ARMC7	633	2286	0.277
FLJ10378	675	2358	0.286
LAG3	700	2115	0.331
ZFPM2	607	1621	0.374
KSP37	602	1595	0.377
NGFR	649	1714	0.379
CXCL1	845	2167	0.390
KEL	729	1587	0.459
KIAA1634	752	1598	0.471

Table S2. Primer pairs for quantitative real-time PCR.

Gene name	Sequence of sense primer	Sequence of antisense primer
ACTB	GAAGTGTGACGTGGACATCC	CCGATCCACACGGAGTACTT
LOC100129973	GAGAGGACGGGAGAGGAGAC	GTGAGGCAGGCGGTGAAG
API5	GTCTTCATTACCATTACCTCTACACTG	GATTATTGCCACCACTCCATAGC
BCL2L12	GCTGTTTGGCACCGACTACC	GGAGGAGACGATGAGGATGAGA
miR-4707-5p	AATAAATAAGCCCCGGCG	ACGCTTCACGAATTTGCGTGTC
miR-4767	AATAATACGCGGGCGCTC	ACGCTTCACGAATTTGCGTGTC
LOC100129973 for RIP	GCTTAGGGATGCTGGGTGATTT	GATCTGCACGAGAGGCTTGG
